# Terahertz Imaging for Paper Handling of Legacy Documents

**DOI:** 10.3390/s21206756

**Published:** 2021-10-12

**Authors:** Min Zhai, Alexandre Locquet, David S. Citrin

**Affiliations:** 1 Georgia Tech-CNRS IRL2958, Georgia Tech Lorraine, 2 Rue Marconi, 57070 Metz, France; zhaimin01@gatech.edu (M.Z.); alexandre@gatech.edu (A.L.); 2 School of Electrical and Computer Engineering, Georgia Institute of Technology, Atlanta, GA 30332, USA

**Keywords:** terahertz spectroscopy, terahertz nondestructive evaluation, terahertz imaging, terahertz dielectric properties, paper handling

## Abstract

Despite predictions of the paperless office, global demand for printing and writing paper remains strong, and paper appears to be here to stay for some time. Not only firms, but also governments, libraries, and archives are in possession of large collections of legacy documents that still must be sorted and scanned. In this study, terahertz-based techniques are demonstrated to address several routine tasks related to the automated paper handling of unsorted legacy documents. Specifically, we demonstrate terahertz-based counting of the number of sheets in unconsolidated paper stacks, as well as locating stapled documents buried in paper stacks.

## 1. Introduction

The concept of the paperless office was proposed in 1975 to address the environmental impacts resulting from paper-product manufacturing [1]. With the rise of electronic documents, expectations of the demise of paper have become commonplace. Although some enterprises and public institutions have completed the transformation to digital documents, global writing- and printing-paper consumption remains strong [2]. According to the latest data published by Ian Tiseo in 2021, the global demand for printing and writing paper was 99 million metric tons in 2018, and is expected to be ∼90 million metric tons by 2030 [3]. The continued reliance on paper demonstrates that the realization of the paperless office is yet to materialize. Reports of the death of paper are greatly exaggerated.

Paper handling, in general, encompasses tasks such as collating, trimming, and finishing documents, but also storing, classifying, sorting, and scanning legacy documents. Unlike preparing new documents, information on legacy documents, such as the page count and paper type, whether it has been stapled, or contains foreign objects, may not be known, strongly affecting the ability to automate such tasks. While page count in new documents is a relatively simple task (based on weight or thickness measurements), stacks of legacy documents may involve paper of various grams per square meter, various sizes, and various types, prohibiting such simple approaches. The loading of such documents into a feeder is also made difficult by the possible presence of tape, staples, and paperclips. Consequently, a number of routine paper-handling tasks still require extensive human intervention. Approaches to automate repetitive labor-intensive tasks in dealing with legacy documents are desired.

The terahertz (THz) portion of the electromagnetic spectrum—by convention ranging over the frequency range ν∈[100 GHz, 10 THz]—has attracted considerable interests for a range of three-dimensional imaging applications on the μm to mm length scales. THz waves can penetrate many electrically insulating materials in a noninvasive and nonionizing manner, which is useful for many practical applications, in comparison with x-rays (ionizing) and infrared imaging (typically not three-dimensional) [4,5]. The reflected THz signals carry *quantitative* information about the interior structure of objects, while the transmitted THz signals can be used as optical and dielectric properties of materials at THz frequencies [6]. While other optical techniques, such as confocal microscopy and two-photon fluorescence microscopy, can provide three-dimensional layered images, they may not be capable of easily handling thick stacks of documents.

Paper is one such material that would appear to be amenable to THz characterization. The baseline dielectric properties of paper in the THz range, *viz.*, the refractive index n(ν) and the attenuation coefficient α(ν), need to be established to enable the implementation of THz techniques for structural characterization. Indeed, the optical constants of various types of papers were measured and investigated quantitatively in Refs. [7,8,9]. In a similar vein, Ref. [10] have inferred paper basis weight via its THz dielectric properties. Because the THz signal is sensitive to the presence of moisture in media, THz techniques have been specifically applied to ascertain water content in paper [7,11]. It should be noted that, although commercial microwave transmission moisture meters that measure the moisture of paper in a non-contact and non-destructive manner have been in use for decades, THz approaches may enable the determination of the three-dimensional moisture distribution within a paper stack due to the relatively short wavelength range in the THz domain. In Ref. [7], the refractive index of copy paper was seen to decrease from 1.49–1.51 to 1.47–1.49 for ν∈[0.2 THz, 1 THz] after drying using a hair dryer for several minutes. In Ref. [12], birefringence attributed to preferential fiber orientation within lens paper and spruce wood was observed. [he machine direction (MD)/cross direction (CD) ratio is a common measure of paper anisotropy which leads to birefringence; we do not address anisotropy in our study. THz time-of-flight tomography (TOFT) (see Appendix A) has also been used to measure the thickness of individual sheets of paper simultaneously with the moisture content [8]. In THz TOFT, a short THz pulse is launched into the sample. Most commonly, the reflected THz signal is mapped out in time. Due to dielectric discontinuities at the interfaces between different layers, the reflected signal is composed of a sequence of time-delayed reflections, or *echoes*, from these interfaces. The time delays contain information on the layer thicknesses and the number of layers can be obtained from the number of such echoes. An entirely different set of applications is to read text on documents due to the contrast between the refractive indices of ink and the paper [9,13]. For example, in Ref. [9], anti-counterfeit features on banknotes including the watermarks, security threads, and optically variable inks, were identified in THz imaging. Moreover, commonly available paper thicknesses make paper of interest to fabricate THz components [14,15].

Our interest here is to explore the potential of THz techniques for various paper-handling tasks. To provide context, though this is not the sole application, in an automated sheet feeder for large-volume scanning, it is useful beforehand to have a sheet count and determine whether any sheets in a feeder stack may be stapled to prevent feeder failure. In this paper, therefore, we focus on obtaining page count *s* and locating foreign objects (e.g., staples) in multipage legacy documents, which are prerequisites for subsequent paper-handling tasks, such as scanning. After obtaining the dielectric properties of paper stacks for ν∈[0.2 THz, 2 THz] in transmission measurements, the internal structure of paper stacks is presented based on TOFT. The page count of and staple location within paper stacks are determined from THz reflection data. Finally, the exact position of stapled sheets buried in a paper stack are determined by THz TOFT.

The sequel of this paper is organized as follows. In Section 2 we outline the experimental approach. Section 3 contains the dielectric characterization of paper stacks in the THz range. In Section 4 we discuss counting sheets of paper in stacks, while Section 5 considers locating stapled documents in a stack. In Section 6, we conclude.

## 2. Experiment and Sample

**Figure 1:**
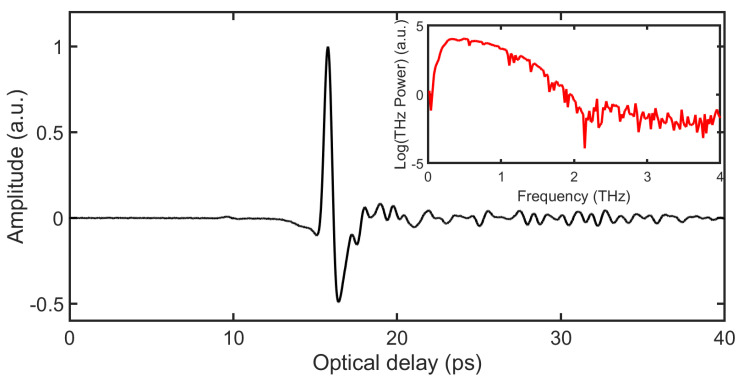
THz reference signal with its frequency spectrum in the inset.

THz transmission and reflection experiments were conducted using a commercial THz time-domain spectrometer (TPS Spectra 3000 from TeraView Ltd., Cambridge, UK) in a laboratory at temperature 22 °C with humidity < 48%. The effective bandwidth of the apparatus is ν∈[60 GHz, 3 THz]; however, to avoid the low signal-to-noise ratio (SNR) at the extreme ends of the band after interaction with the paper stacks, only ν∈[0.2 THz, 3 THz] is used for analysis. Because of confounding effects of water vapor, the propagation path was purged with dry N${}_{2}$ prior to the transmission measurements. Each recorded signal was averaged 1800 times to achieve high SNR. Balancing the noise level with the spectral resolution, the Black-Harris 3-term apodization method was also utilized to smoothen the spectrum and suppress spectral artifacts [16]. For the THz TOFT measurements (in reflection), the sample is moved in discrete spatial steps of 200 μm in the *x*- and *y*- directions by a set of motorized stages. Dry N${}_{2}$ was also utilized prior to each reflection measurement to remove moisture proximate to the THz emitter and receiver. Before imaging the paper stack, a THz reference signal, shown in Figure 1, was recorded by setting a metal plate at the sample position. The result, then, is a three-dimensional dataset with the two transverse directions as two dimensions and the time delay of the measured THz signal as the third dimension.

**Figure 2:**
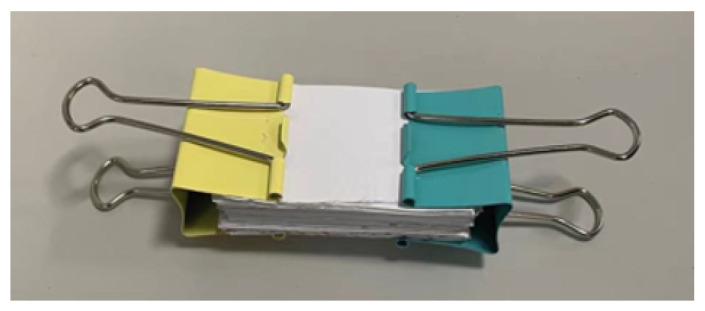
Paper stack held in custom-build jig based on binder clips (Home Depot, medium, multicolor). Size of paper sheets is ∼8 cm × 5 cm.

The paper studied here is standard A4 copy paper (Paperbox brand multifunction white laser-printer and photocopy paper) manufactured by LECTA, Milton Keynes, UK, and the relevant physical properties of the copy paper are shown in Table 1. All paper investigated was stored under the temperature and humidity conditions of the laboratory for which the moisture content of the paper is approximately 7.5% [17]. While individual sheets of paper may have slightly different water content and may otherwise differ, as we shall see below, in THz TOFT experiments, we are still able to identify the various sheets of paper within a stack. We specifically studied unconsolidated paper stacks (s∈[1,155]), i.e., the paper sheets were not compressed together except at the ends away from the scanning area. Therefore, the paper stacks were comprised of copy paper with naturally occurring air gaps between the sheets, presenting a stratified medium. Stacks of paper were cut down to 8 cm × 5 cm rectangles from full A4 sheets (21 cm × 29.7 cm) before being stacked. The ends of the paper stacks were held in a custom-built jig based on binder clips, but the regions of the stacks imaged were not compressed, as shown in Figure 2.

## 3. THz Dielectric Properties

We begin by characterizing the dielectric properties of paper stacks in transmission experiments. In this case, a THz pulse is propagated through the paper stack located at the focus of the optical system, and the effect on the various spectral components is compared with the reference signal (i.e., the THz pulse produced by the apparatus).

**Figure 3:**
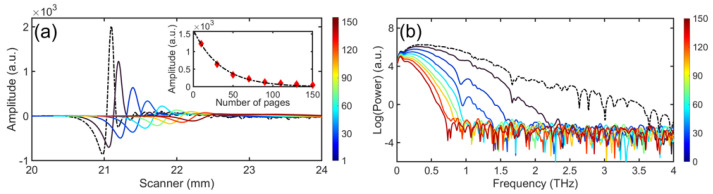
(**a**) THz signals (electric field) transmitted through paper stacks with various page count *s* ranging from 1 to 155. Inset shows the peak amplitude as a function of *s* in the paper stacks. (**b**) Power spectra of the corresponding transmitted signals shown in (**a**). The color bars indicate the number *s* of sheets in a stack corresponding to a given curve.

Figure 3a shows transmitted signals for paper stacks with various page counts *s* ranging from 1 to 155 sheets, and (b) the corresponding power spectra of these transmitted signals. The number *s* of sheets in the stack is coded by the color bar to the right of each panel. It is unsurprising that the reference signal has the highest amplitude and shortest propagation time while both propagation delay and reduction of peak height increase with *s*. Referring to Figure 3b, the useable bandwidth decreases with increasing *s* as the high frequency portion of the spectra fall below the noise floor of the experiment, demonstrating the loss of high-frequency information as the THz waves suffer attenuation in the paper stacks. When s≳155, the transmitted signal amplitude is comparable to the noise. The observed attenuation is in line with that seen in Refs. [8,15], where wave plates of 150 to 220 sheets and intervening air gaps were fabricated.

The refractive index n(ν) and attenuation coefficient α(ν) are calculated based on the complex fast Fourier transform of pulse transmitted through the paper stack compared with the incident (reference) pulse. Denote the amplitude and phase of the electric field as $E_{s}\left(\nu \right)$, $\phi_{s}\left(\nu \right)$ and $E_{r}\left(\nu \right)$, $\phi_{r}\left(\nu \right)$. One obtains [16]

(1) $$\begin{array}{ccc} \alpha (\nu ) & = & -2/d_{s}\ln\left[E_{s}\left(\nu \right)/\left(T\left(\nu \right)E_{r}\left(\nu \right)\right)\right], \end{array}$$

(2) $$\begin{array}{ccc} n(\nu ) & = & 1+c\left[\phi_{s}\left(\nu \right)-\phi_{r}\left(\nu \right)\right]/\left(2\pi \nu d_{s}\right) \end{array}$$

where $d_{s}$ is the paper-stack thickness measured using calipers with an accuracy of 10 μm, and T(ν) is the transmittance associated with the air/paper stack and paper stack/air interfaces,

(3) $$T\left(\nu \right)=1-{\left[n\left(\nu \right)-1\right]}^{2}/{\left[n\left(\nu \right)+1\right]}^{2}.$$

The paper stack, of course, also contains air gaps (unit refractive index) between adjacent sheets, leading to a discrepancy between $d_{s}$ and sd with *d* the single-sheet thickness (102 ± 4 μm). In measuring $d_{s}$, the calipers were tightened to remove excessive air from the paper stack without unduly compressing the paper. Though more accurate approaches may be implemented, this provided consistent results as we see below. Moreover, as a multilayer structure, coherent interference effects (due to multiple reflections) will play a role in the reflected signal. Assuming multiple reflections can be neglected in the THz transmission, only the paper contributes to n(ν) since the refractive index of air is 1. This assumption is justified due to the rather modest value of n(ν) for paper (see below).

**Figure 4:**
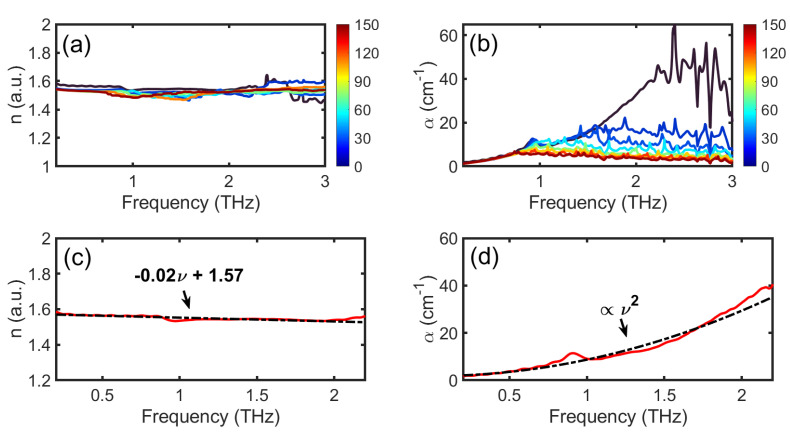
(**a**) Refractive index n(ν) and (**b**) attenuation constant α(ν) of paper stacks with *s* = 1 to 155. (**c**) n(ν) for *s* = 10 (by way of example, solid line in red) and a linear fit (dash-dotted line in black). (**d**) α(ν) for *s* = 10 (solid line in red) and a quadratic fit (dash-dotted line in black).

The frequency-dependent optical constants n(ν) and α(ν) are plotted in Figure 4 with (a) n(ν) and (b) α(ν) for s∈[1,155]. The optical constants are meaningful for ν within the effective dynamic range determined by *s* as discussed above (see Figure 3b). The dispersion dn(ν)/dν<0 for all cases, and n(ν) decreases from 1.59 at 0.2 THz to 1.54 at 2 THz when s=10, as shown in Figure 4c; dn/dν≈−0.02 THz${}^{-1}$. The values are in good agreement with those reported in Ref. [7]. The weak dispersion in n(ν) along with the ν-dependent α(ν) contributes to the change in pulse shape during the propagation in the paper stack. Figure 4b shows α(ν) for various *s*, and α(ν) increases from 1.7 cm${}^{-1}$ at 0.2 THz to 32.7 cm${}^{-1}$ at 2 THz for *s* = 10 sheets. The values of α(ν) obtained in this work are consistent with literature values [7]. The values plotted are only meaningful for ν less than an upper value determined when the power spectra in Figure 3b drops below the noise floor. Referring to Figure 4b, this maximum ν decreases with increasing *s* and corresponds to the maximum frequency for which α(ν) is quadratic. For higher frequencies, α(ν) is dominated by noise. Thus, the apparent low attenuation at high ν for large *s* is just an artifact of this *s*-dependent dynamic range. α(ν) within the effective dynamic range [0.2 THz, 2.2 THz] (for s=10), estimated based on Figure 3b, varies roughly quadratically in ν, as shown in Figure 4d. Similar behavior of α(ν) was also found in Ref. [7] and is typical of disordered media [6,18,19,20,21].

## 4. Page Count *s* in Paper Stacks

The first practical application of the THz techniques discussed here is to ascertain page count *s* from the THz data. As observed in the inset of Figure 3a, the maximum amplitude of the transmitted signal through the paper stack decreases approximately exponentially with increasing *s*. We can thus infer *s* roughly by calibrating the transmitted amplitude against *s*. Such an approach gives rise to an error of a few sheets, increasing with *s*. It must also be pointed out that this technique only works when the paper in the stack is of a uniform and known type. For the cases we studied, when s≤60, the error between the inferred page count and the nominal *s* is ≤4, while the error reaches 30 when s=150 based on calibrating *s* against transmission. To extend this approach to larger *s* would require more powerful THz sources and lower-noise THz measurements.

To circumvent the limits imposed by error in the above approach as well as the requirement of working with a single known paper type, we employ an alternative approach based on THz TOFT. This approach will produce essentially error-free measurements of *s* for s≲20 and can provide additional structural information on paper stacks. Namely, we employ THz TOFT to reconstruct the stratigraphy of the paper stack. Due to the presence of noise in the measurement, we implement frequency wavelet-domain deconvolution (FWDD) to enhance the signal-to-noise ratio (SNR) in the measurement. FWDD, as a simple but effective denoising technique, and involves cascading a band-pass filter with a filter based on wavelets that resemble the incident THz pulse. The wavelet basis selectively filters out unrelated features from the detected signal retaining those features that resemble the incident THz (reference) pulse (see Appendix A). Because dispersion shown in Figure 4c is weak (and within the frequency range that dominates the signal, the frequency dependence of α(ν) is not substantial), FWDD is easily implemented.

**Figure 5:**
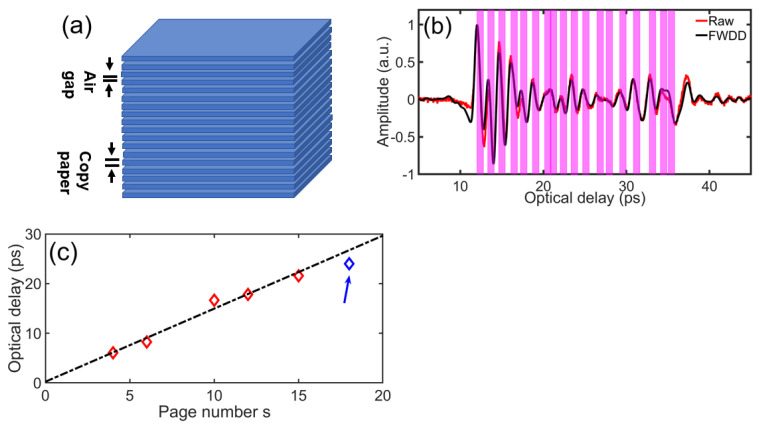
(**a**) Schematic of *s* = 18 stack. (**b**) Raw (red) reflected signal in THz TOFT experiment and the corresponding deconvolved signal after frequency wavelet-domain deconvolution (FWDD) (black). Pink vertical bars indicate the reconstructed locations of the paper sheets in the stack. (**c**) Relationship between optical delay from the linear fit based on the data for *s* = 4, 6, 10, 12, and 15 (red) and the measured optical delay (blue) for *s* = 18.

We illustrate the results for s=18 in Figure 5. Figure 5a shows a schematic diagram of an s=18 stack, while (b) shows the corresponding raw reflected signal from the THz TOFT experiment in red and the FWDD reconstruction in black. FWDD is helpful to clean up the signal to unambiguously track the various echoes. The approach to estimate *s* is to count the number of reflections (one from each side of each paper sheet) in reconstructed THz TOFT signal. Associated with each sheet of paper is a positive-amplitude echo (from air to the higher-index paper) and a negative-amplitude echo (from higher-index paper to air). The pink vertical bars in Figure 5b indicate the reconstructed locations of the paper sheets. Between successive sheets of paper are air gaps. Inasmuch as the sheets in the stack are not compressed during the measurement, the air-gap thicknesses vary between sheets. In addition, the surface texture may contribute to formation of the airgaps; it would be interesting to see if this occurs with smoother paper, e.g., publication-grade lightweight coated paper or glossy newsprint. Even though we do not fully characterized the air gaps between the sheets, because the refractive index of air is unified, the relative optical delay within paper stacks compared with an equal overall thickness of air is roughly proportional to *s* (for a uniform paper type as herein) as shown in Figure 5c, in which the experimental points for *s* = 4, 6, 10, 12, and 15 are also shown. The error between the estimated page count based on the measured optical delay using the linear relationship fitted by the data for *s* = 4, 6, 10, 12, and 15, and the nominal value for *s* = 18 is smaller than 1. Of course, the error is reduced by looking directly at the stratigraphic reconstruction.

## 5. Three-Dimensional Staple Location

The handling of unsorted legacy documents may be complicated by foreign objects hidden in paper stacks. For example, loose staples or paper clips may be located between sheets of paper, and some pages within a stack may be stapled, and these staples may not be visually evident. In this section, we demonstrate the three-dimensional location of stapled documents hidden in paper stacks using the technique of THz TOFT.

**Figure 6:**
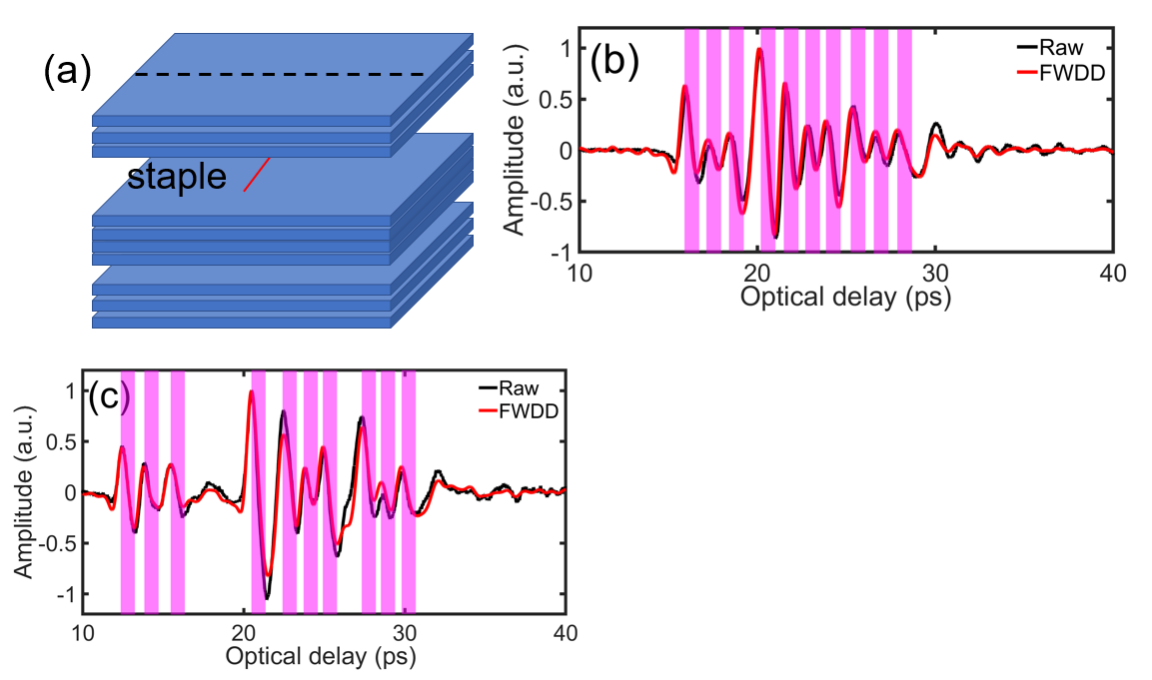
(**a**) Schematic of *s* = 10 stack with the four central pages stapled together. Raw (black) reflected signal, and FWDD (red) reconstructed signals in THz TOFT data (**b**) away from the staple, and (**c**) near the staple, respectively. The horizontal dashed line near the center of paper stack *s* = 10, marked ‘cross-section,’ refers to the B-scan presented in Figure 7a,b.

We consider an s=10-sheet stack with the central four sheets stapled together, as shown schematically in Figure 6a. The raw (black) reflected THz TOFT signals away from and near staple, as well as the corresponding deconvolved signals by FWDD (red) are shown in Figure 6b,c, respectively. Not surprisingly, near the staple, relatively large air gaps just above and below the four stapled sheets are clearly seen, and the four stapled sheets themselves are compressed together, as evident in Figure 6c.

**Figure 7:**
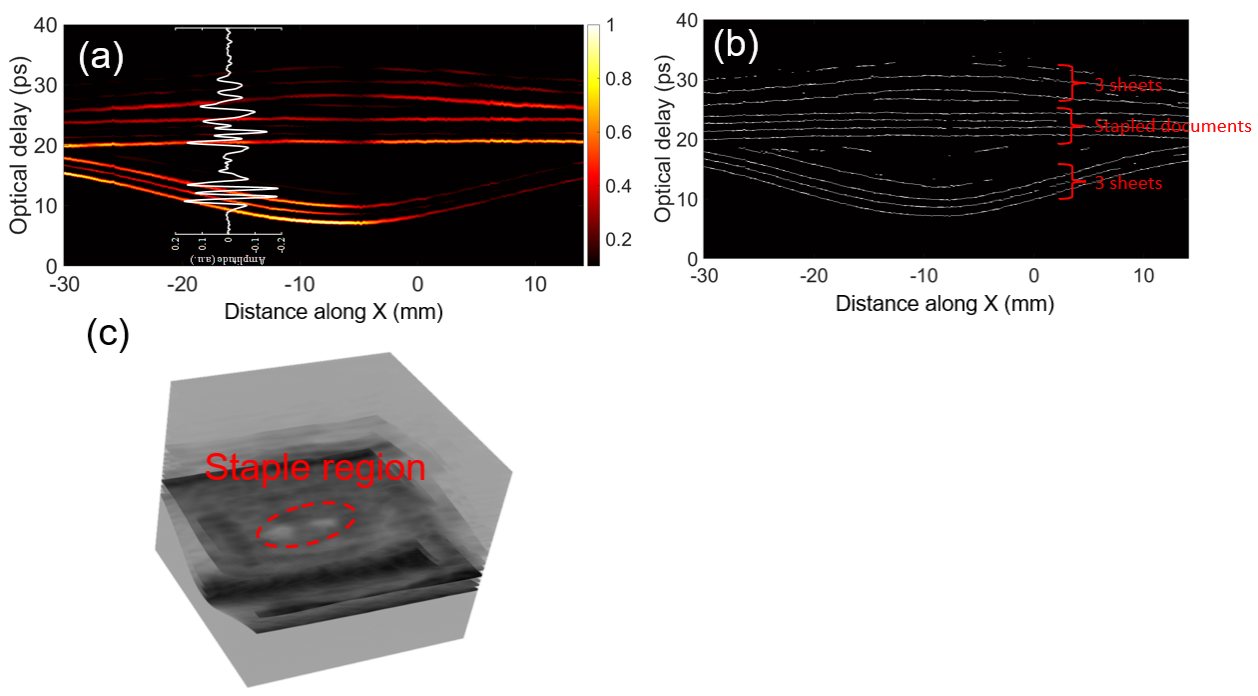
(**a**) B-scan through a cross section through the s=10 stack containing a stapled document along the dashed line shown in Figure 5a with an example of the FWDD reconstructed signal at the position indicated. Large air gaps above and below the stapled sheets are evident. The color scale is linear in the amplitude of the reflected THz signal. Thus, yellow indicates a strong reflection, red a moderate reflection, etc. (**b**) shows the corresponding binary THz B-scan based on the FWDD reconstruction. (**c**) Three-dimensional rendering of THz TOFT data to emphasize the staple location.

To better visualize the structure, we present the FWDD reconstructed data in a cross section through the paper stack, known as a B-scan in Figure 7a. A B-scan is a cross section in space through the three-dimensional dataset of the reflected THz signal, i.e., in the two transverse directions across the sample and for the third dimension, the time delay of the detected reflected THz signal serves as a surrogate for depth into the sample in the axial direction. (To emphasize, we indeed have a three-dimensional dataset and are free to choose any cross section; we here choose a cross section that is particularly illustrative of the approach.) Figure 7a also shows an example of the FWDD reconstructed THz TOFT signal at the position −20 mm on the cross section. Note that the B-scan is simply composed of all such signals along the cross section plotted on the color scale indicated on the right.

In order to eliminate the influence of the alternating signs of the successive echoes as well as the variation in the amplitudes of the peaks/valleys in the reflected THz signal, we can replot the B-scan in Figure 7a based on the FWDD reconstruction as a binary B-scan in which a valid extremum plotted as a white point against a black background regardless of the sign and amplitude of the peak/valley, as shown in Figure 7b. The three loose sheets above and the three loose sheets below the four stapled sheets, as well as the stapled sheets, are clearly seen as indicated. To further facilitate visualization of the staple location, we present a three-dimensional rendering, based on FWDD reconstructed data, in Figure 7c. Here, the reflection from the legs of the staple are found. Of course, this rendering can be freely rotated on the computer assisting in the visualization. Note that metals present strong THz reflections whether or not the metal is ferrous; thus, THz TOFT can detect, for example, aluminum staples, whereas simple magnetic approaches are limited to iron-bearing staples. Magnetic approaches are also likely to have poor resolution. In our experiments, the width of the wire forming the staple itself may be close to barely resolved as the width of the beam at the focus at 2 THz is ∼700 μm in air for our numerical aperture. Finally, though x-rays are expected to clearly identify staples in paper stacks, ionizing radiation is likely to be avoided due to potential health risks.

## 6. Conclusions

We apply THz techniques for several routine tasks related to paper handling of legacy documents. We first characterize the dielectric properties of stacks of standard copy paper in the band ν∈[0.2 THz, 2 THz] in transmission, and find that there is a detectable THz signal through paper stacks with page count up to s=155. We obtain the refractive index n(ν) and the attenuation constant α(ν) as functions of frequency for ν in the band mentioned above. The incident THz signal is attenuated by an amount depending on the page count *s* in the stack. This suggests a transmission measurement of *s* calibrated based on detailed measurement of the transmitted THz pulse amplitude through stacks with various *s*. Error is <4 when s≤60 and reaches 30 when s=150. This approach is rapid and relatively easy; however, the main drawback of such an approach only works well for a single paper type and must be calibrated in advance for that type of paper.

We next turn to an alternative approach to ascertain *s*, namely THz TOFT. Here, the idea is to reconstruct the stratigraphy of the paper stack presented based on the raw THz TOFT data in conjunction with FWDD. The error in *s* is less than one sheet when s≲20; however, the error increases proportional to *s* and exceeds unity when s≳20. For thicker stacks, other noise-mitigation strategies, either in the physical measurement or in the signal processing, may increase the number of sheets above 20 for which the error in *s* remains less than one. In addition, we have not accounted in our analysis for dispersion in n(ν) or α(ν), which eventually limits the effectiveness of FWDD. Stratigraphic reconstruction approaches enabling the inclusion of dispersion have been developed [22,23]. Still, it is noteworthy that this approach is not constrained by paper type and can deal with mixed paper.

The next application discussed is the detection of foreign objects in paper stacks. We use THz TOFT to detect a staple binding the four central sheets in a s=10-sheet paper stack, even though it still works for s=18 paper stacks. The staple is located successfully in three-dimensions. Not shown in this paper, we have also been successful in locating loose staples in paper stacks as well.

The examples demonstrated here open the door to other related applications. Though the examples we present here focus on a single paper type, legacy documents might also have mixed paper. Other types of foreign objects, including paper scraps, self-adhesive notes, tape, paperclips—both metal and plastic—might also be present. These situations are yet to be studied. Nonetheless, our work suggests that THz techniques merit further exploration for practical implementation in paper handling of legacy documents.

## Figures and Tables

**Table 1:** The physical characteristics of the copy paper studied.

Properties	Value	Tolerance
Weight	80 g/m${}^{2}$	±4%
Thickness	102 μm	±4%
Opacity	87%	±4%
Relative humidity	40%	±5%

## Data Availability

The datasets generated and/or analysed during the current study are available from the corresponding author on reasonable request.

## Appendix A. Reconstruction Algorithms

• THz TOFT: The technique to reconstruct the stratigraphy using THz pulses in the time domain is based on time-of-flight techniques (TOFT). In short, when THz pulses impinge on a stratified medium, owing to the discontinuities in refractive index at each interface, specular reflected pulses or *echoes* arise at the interfaces between various layers. These echoes arrive at the detector with various time delays due to the finite speed of light at which the THz signals propagate. The stratigraphy of the sample can be quantitatively reconstructed based on the time delays between the echoes together with a knowledge of the refractive indices of the constituent materials. More detailed information can be found in Refs. [24,25].

• FWDD: Frequency wavelet-domain deconvolution (FWDD) is a technique to construct a filter that selectively passes features resembling those of the incident pulse, since the legitimate echoes will also look like the incident pulse. One seeks to extract the impulse response function of the sample by first employing filters to suppress noise at low- and high-frequencies. Then further enhancement to the signal-to-noise ratio is achieved by the actual wavelet-denoising. More detailed information can be found in Refs. [26,27].
